# Chemical Elements and Thickness of *Candida albicans* Biofilm Induced by Glucose, Lactose, Protein, and Iron

**DOI:** 10.1055/s-0045-1808261

**Published:** 2025-05-20

**Authors:** Indah Listiana Kriswandini, Sidarningsih Sidarningsih, Prawati Nuraini, Nur Mega Dwi Y., Mohammed Ahmed Aljunaid, Oki Fadhila

**Affiliations:** 1 Department of Oral Biology, Faculty of Dental Medicine, Universitas Airlangga, Surabaya, Indonesia; 2 Departement of Pedodontics, Faculty of Dental Medicine, Universitas Airlangga, Surabaya, Indonesia; 3 Department of Oral and Dental Medicine, Faculty of Medicine, Taiz University, Taiz, Yemen

**Keywords:** health, biofilm thickness, *Candida albicans*, chemical elements, CLSM

## Abstract

**Objective:**

Health is the most important aspect that needs to be considered, and the oral cavity cannot be separated from other parts. *Candida albicans* is a normal flora in the oral cavity that is a major cause of oral candidiasis. Research on biofilms can help prevent oral candidiasis infection in the community. Biofilms are involved in the pathogenesis and could be examined using an electron and fluorescence microscope, which can analyze the whole biofilm in actual conditions. This study aims to determine the chemical elements and thickness of *Candida albicans* biofilms induced by glucose, lactose, soy protein, and iron.

**Materials and Methods:**

This analytic observational study was carried out by observing the chemical elements and thickness of the biofilm by scanning electron microscopy with energy dispersive X-ray (SEM-EDX) and confocal laser scanning microscopy (CLSM). SEM-EDX data analysis used the EDAX APEX software and CLSM used the Olympus FluoView ver 4.2.a.

**Results:**

SEM-EDX examination showed the formation of *Candida albicans* biofilm induced by glucose, lactose, soy protein, and iron with similarity in the percentage of the most constituent chemical elements, namely, oxygen, carbon, nitrogen, and phosphorus, and the least were sulfur. The thickest biofilm was found in the induction of iron, glucose, and lactose, and the thinnest was soy protein.

**Conclusion:**

The chemical elements of *Candida albicans* biofilm induced by four different inducers has the same percentage of the composition of elements, namely, oxygen, carbon, nitrogen, and phosphorus, and the least were sulfur and the thickest biofilm was by the induction of iron, glucose, and lactose, and the thinnest was by soy protein.

## Introduction

Health is the most important aspect that needs to be considered and the body is a unified system, and as such the oral cavity cannot be separated from other parts. 1 Candidiasis is a fungal infection that often attacks the oral mucosa. Oral candidiasis is an infection caused by *Candida albicans* , which is the normal flora of the oral cavity in 30 to 50% of individuals. 2 *Candida albicans* can change its morphology from yeast cells to hyphal form, which is the core of pathogenesis, thus increasing the ability of *Candida albicans* to form biofilm. 3

*Candida albicans* can form biofilms better at acidic pH than normal or alkaline pH. The formed biofilm consists of cells covered by extracellular matrix and plays a role in stabilizing the interaction of cells with the attachment surface. 3 4 Biofilms are a collection of microorganisms that are firmly attached to a surface along with organic material and are enveloped by an extracellular polymer matrix released by the microorganism. Cell attachment produces a high hydration matrix consisting of exopolysaccharides, extracellular deoxyribonucleic acid, proteins, and lipids. Biofilms are medically important because they are involved in the pathogenesis of microorganisms to cause infectious diseases. 5 6

Biofilm formation can be divided into four stages, namely, reversible cell adhesion, irreversible adhesion, biofilm maturation, and cell dispersion. 7 Biofilms generally form 80 to 85% extracellular polymeric substances (EPS) and only 15 to 20% are cells as they go along the process of cell maturation and dispersion. EPS formed has a variety of sizes, composition, and chemical properties produced by microorganisms and play a role in the process of adaptation, resistance, and functional cells to the environment. 8

Some materials such as glucose, lactose, soy protein, and iron have an influence in the formation of biofilms. Research states that the process of glucose intervention, as a single monosaccharide or lactose constituent, occurs in the process of glucan synthesis through the conversion of glucose into uridine diphosphate-glucose with the help of certain regulators, resulting in an increase in the thickness of the biofilm matrix. 9 Previous research revealed that the content of soybean played a role in increasing the colonization seen in macroscopic observations. 10 11 Some previous studies said iron could influence the formation of biofilms in addition to their role in the development and virulence of microorganisms. 12

Previous research on *Candida albicans* used an enzyme-linked immunosorbent assay reader to measure biofilm thickness and sodium dodecyl sulfate-polyacrylamide gel electrophoresis to express molecular weight through the number of protein bands formed. A limitation in earlier studies is the absence of a visible composition of the chemical elements constituting proteins in biofilms. Therefore, further research utilizing scanning electron microscopy with energy dispersive X-ray (SEM-EDX) and confocal laser scanning microscopy (CLSM) examinations is necessary.

SEM and CLSM examination can be used to study the morphology of the biofilm matrix from *Candida albicans.* The use of CLSM combined with fluorescence material is an effective tool for analyzing biofilm as a whole under real conditions. This study aims to determine the chemical elements and thickness of *Candida albicans* biofilm induced by glucose, lactose, soy protein, and iron through SEM-EDX and CLSM examination. This research is expected to find scientific evidence related to chemical elements and biofilm thickness that can be used as a reference in the preparation of prevention measures for oral cavity infections in the community and the development of disease detection kits for oral candidiasis infection with biofilm indicators.

## Material and Methods

### Bacteria Sample and Ethical Clearance

The bacteria sample was *Candida albicans* from the Research Center Institute of the Faculty of Dental Medicine, Universitas Airlangga University with a sample size of one for each inducer. The approval of this study was obtained by the Ethics Commission of the Faculty of Dental Medicine, Universitas Airlangga, Surabaya, Indonesia (Reference number 382/HRECC.FODM/VI/2019).

### *Candida albicans* Biofilm Culture

The research process was divided into two parts, for SEM-EDX and CLSM examinations. The culture process was based on the Akay et al 13 procedure by aerobic culture of *Candida albicans* stock in 10 mL of Brain Heart Infusion Broth (BHIB) for 24 hours (McFarland standard 5). Each culture was induced with 0.5 mL 5% glucose and 5% lactose, 5 mL 5% tryptic soy broth (TSB), and 0.2 mL 2% iron, and was recultured for 24 hours. The biofilm formed was centrifuged for 10 minutes at a speed of 3,000 revolutions per minute and the BHIB media was removed then rinsed with phosphate buffer saline (PBS) twice to remove its planktonic cells. The precipitate was mixed with glutaraldehyde (GA) in PBS and transferred to the microtube. CLSM examination was carried out by transferring *Candida albicans* culture to microplate with coverslips and given inducer material for further incubation for 24 hours.

### SEM-EDX Examination

Biofilms were fixed with 2% GA for 2 to 3 hours at 4°C. Samples were washed with PBS pH 7.4 three times every 5 minutes at 4°C. The solution is replaced with osmic acid 1% for 1 to 2 hours at 4°C. Then washed again and dehydrated with multilevel alcohol respectively for 15 to 20 minutes at a concentration of 30, 50, and 70% at 4°C, followed by a concentration of 80 and 90%, absolute 2× at room temperature. The solution is replaced with amyl acetate and dried with a critical point drying tool. Sample was attached to the holder using specific glue. Sample coating with pure gold was carried out using a vacuum evaporator. SEM examination uses 1500× magnification with the EDAX APEX software.

### CLSM Examination

Cultures in 4 wells (800 μL each) were induced with 40 μL of 5% glucose solution, 5% lactose, 5 mL 5% TSB, and 16 μL of 2% iron and were incubated aerobically for 24 hours. BHIB is removed and rinsed with PBS three times each for 10 minutes. The procedure was carried out based on the research of Vila et al 14 by fixing samples using 2 mL of 4% paraformaldehyde for 20 minutes then discarded. Samples were given concanavalin A-fluorescein isothiocyanate conjugate (ConA-FITC) (Sigma Aldrich) stain as much as 400 µL, homogenized, and allowed to stand for 15 minutes in a dark room. The stain material is discarded and given a 400-μL propidium iodide (PI) (Biolegend) counter stain in the same way. The stain material is removed, and the sample is transferred to a glass object to be observed with CLSM and analyzed using the Olympus FluoView ver 4.2a software.

## Results

### SEM-EDX Results

**Fig. 1:**
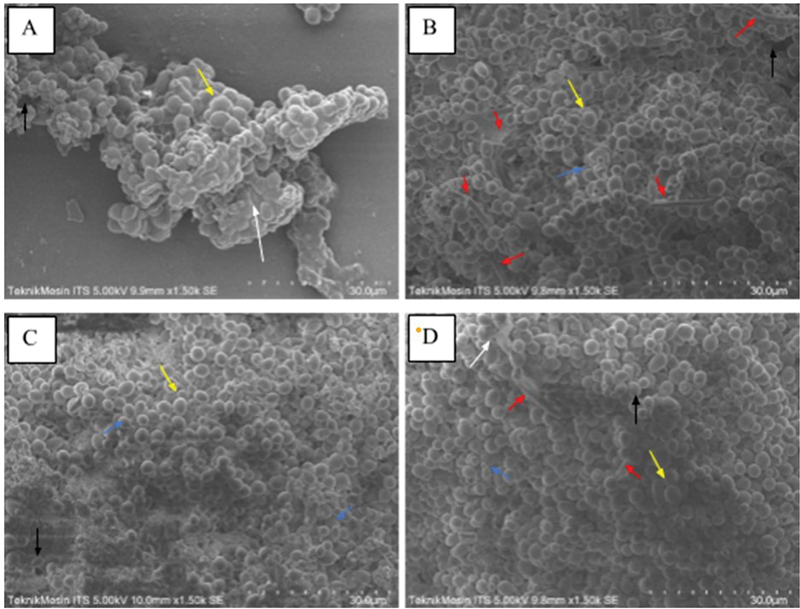
Scanning electron microscopy (SEM) images of biofilm induced by ( **A** ) glucose, ( **B** ) lactose, ( **C** ) soy protein, and ( **D** ) iron. The arrow color indicates: yeast cells (yellow arrow), yeast cells (yellow arrow), channel (black arrow), extracellular polymeric substances (EPS) (white arrow), and pseudohifa (red arrow).

**Fig. 2:**
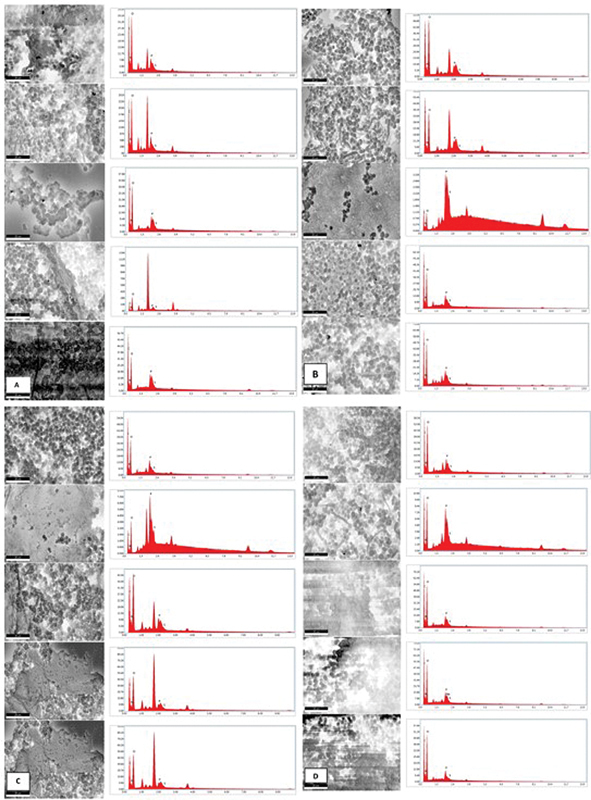
Energy dispersive X-ray (EDX) graph chemical elements of *Candida albicans* biofilm induced by ( **A** ) glucose, ( **B** ) lactose, ( **C** ) soy protein, and ( **D** ) iron.

The scanning results using SEM ( Fig. 1 ) show a surface picture of *Candida albicans* biofilm induced by glucose, lactose, soy protein, and iron with a magnification of 1,500× to get a picture of cells and EPS matrix. SEM images of biofilm induced by glucose, lactose, soy protein, and iron have the most formation in the form of yeast cells (yellow arrow) and a small portion of blastoconidia (blue arrows). Another formation was seen in the form of a channel (black arrow) on all induced biofilms. Induced biofilms have an overall composition of the same cell constituents, but there is a difference in glucose induction, which shows a large aggregate of candida cells and a solid (visibly contrasted/bright) EPS (white arrow) layer on glucose and iron induction. Biofilms with lactose induction appear to have more pseudohifa (red arrow) formations compared to glucose, soy protein, and iron induction biofilms. The SEM image results obtained were analyzed using EDX chemical elements to obtain the average percentage of the chemical elements C (carbon), N (nitrogen), O (oxygen), P (phosphorus), and S (sulfur) from the examination results at five random locations for each inducer (%). Fig. 2 illustrates the overall of EDX graph chemical elements of *Candida albicans* biofilm induced by glucose, lactose, soy protein, and iron. The EDX graph shows the *Candida albicans* biofilm induced by (A) glucose, (B) lactose, (C) soy, and (D) iron. The results in Table 1 show that the biofilms induced by each inducer have the same higher composition of elements by oxygen, followed by carbon, nitrogen, phosphorus, and the least is sulfur.

**Table 1:** The average of each chemical element in the *Candida albicans* biofilm induced by glucose, lactose, soy protein, and iron

	Chemical elements (%)				
	C (carbon)	N (nitrogen)	O (oxygen)	P (phosphorus)	S (sulfur)
Glucose	30.71	15.45	47.60	4.85	1.38
Lactose	33.83	15.44	39.07	7.66	3.99
Soy protein	30.97	15.24	43.15	7.76	2.88
Iron	32.51	15.60	45.40	4.51	1.98

### CLSM Results

**Fig. 3:**
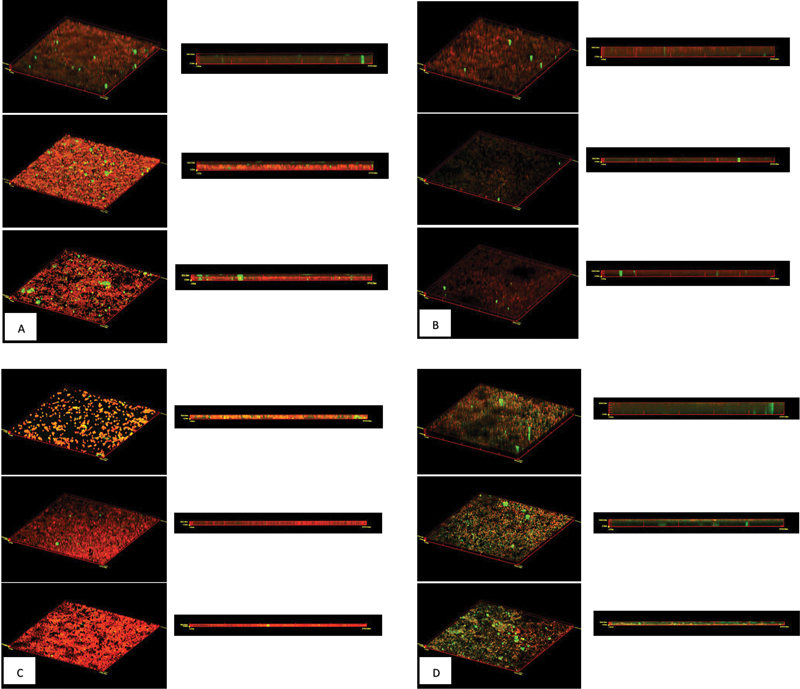
Three-dimensional (3D) visualization of *Candida albicans* biofilm induced by ( **A** ) glucose, ( **B** ) lactose, ( **C** ) soy, and ( **D** ) iron.

CLSM examination results are seen based on the average fluorescence intensity at three random locations shown by ConA-FITC as polysaccharide dyes and PI as *Candida albicans* cell dyes in arbitrary unit. Combination of fluorescence intensity produced by staining with ConA-FITC and PI shows a three-dimensional (3D) visualization of *Candida albicans* biofilm induced by (A) glucose, (B) lactose, (C) soy, and (D) iron, so that the biofilm thickness can be measured and expressed in nanometer units ( Fig. 3 ). The results in Table 2 show the fluorescence intensity of biofilm induced by glucose, lactose, and soy protein have a higher PI (cell) intensity than the intensity of ConA-FITC (polysaccharides), in contrast to biofilm induced by iron that have lower PI intensities compared to the intensity of ConA-FITC. The thickest biofilm thickness was found in the induction of iron, glucose, and lactose, and the thinnest was soy protein.

**Table 2:** The average results of fluorescence intensity and thickness of *Candida albicans* biofilm induced by glucose, lactose, soy protein, and iron

	Fluorescence intensity (AU)		Thickness (nm)
	ConA-FITC	PI	
Glucose	425.06	843.62	12.33
Lactose	229.13	243.66	12.00
Soy protein	309.76	579.59	7.00
Iron	403.18	332.98	13.00

Abbreviations: AU, arbitrary unit; ConA-FITC, concanavalin A-fluorescein isothiocyanate conjugate; PI, propidium iodide.

## Discussion

SEM examination shows that by induction of glucose, lactose, soy protein, and iron, *Candida albicans* can form biofilms. According to Koo and Yamada, 15 the formation of biofilms depends on environmental conditions that are influenced by the concentration of pH, O _2_ , signaling molecules, and other substances as sources of their metabolism. This research produces a biofilm SEM picture showing the presence of a microcolony structure separated by a cavity in the form of a channel. Cells that have channels and pores on the biofilm allow an increase in influx of substrates and nutrients to the inside of the biofilm as well as an efflux pathway for metabolic waste products. 16

Glucose-induced biofilm show a large aggregate of candida cells trapped in the EPS matrix because *Candida albicans* easier to use monosaccharides such as glucose so that it can significantly increase cell numbers in the early stages of biofilm formation. 17 Biofilms with lactose induction have more morphology in pseudohifa cells because lactose induces an increase in the expression of the hyphal wall protein 1 (HWP1) gene that plays a role in hypha formation from planktonic cells. 18 Biofilms with soy protein induction using TSB show the formation of filaments, which may be caused by the phosphorus component in TSB. 19 Biofilms with induction of iron is carried out using 2% iron solution because higher concentrations in previous studies caused candida cells to die. Biofilms with low levels of iron induction increase cell surface hydrophobicity so that the collection of adorable cells increases and biofilms are formed that are denser and resistant to mechanical interventions. 20 Biofilm samples that have been subjected to scanning will be analyzed by EDX to determine the percentage of chemical elements in carbon, nitrogen, oxygen, phosphorus, and sulfur because they are some of the components that are likely to make glycoproteins in biofilms. 21

*Candida albicans* can use a variety of carbon sources derived from glucose, nonfermented sugar, fatty acids, and amino acids. The main nitrogen sources such as ammonia, glutamine, asparagine, and glutamate as well as alternative nitrogen sources such as amino acids, polyamines, and protein hydrolysate can also be used by *Candida albicans* . 22 The ability of *Candida albicans* to utilize various types of material sources is likely to cause the results of EDX analysis to show the percentage similarity composition of the chemical constituents of biofilms.

The biofilm EPS formed functions as a regulator and cellular signal to maintain the biofilm architecture by providing 3D microenvironment resulting in the formation of chemical and nutritional substances. The environment produces a variety of concentrations of pH, O _2_ , signaling molecules, and various other materials, as such it can be said that the formation of EPS is influenced by environmental nutrients, which also affect the formation of glycoproteins, carbohydrates, lipids, and nucleic acids. 15 16

Utilization of oxygen in *Candida albicans* centers on the electron transport chain in the mitochondria where oxygen plays an important role as electron acceptors in the formation of chemical energy through the process of mitochondrial respiration. 23 Carbon plays a major role in the formation of biofilms, antifungal resistance, virulence factors, and cell wall structure, which determines the pathogenicity of *Candida albicans* . Induction of biofilms with high glucose levels can increase cell adherence and biofilm formation in the oral cavity of the host. 17 The main and alternative nitrogen source can be used by *Candida albicans* in metabolism because it can secrete large amounts of secreted aspartyl protease (SAP) enzymes that can change proteins to become peptides and amino acids, which are then carried by special transporters. SAP2 and GAP2 gene expression will be induced so that it can increase the adhesion, growth, and formation of the biofilm. 24 Phosphorus metabolism in the form of phosphate in a number of microorganisms is important to increase the virulence and the number of pathogenic microorganisms. Low phosphate levels cause the accumulation of PHO4 in the nucleus thereby activating several genes that are sensitive to small amounts of phosphate. Research on the growth of *Candida albicans* shows the growth of candida filaments that grow on media with low phosphate levels. 19 Sulfur is another important nutrient needed by fungi because it is part of the proteogenic amino acid cysteine and methionine and important organic molecules such as coenzyme-A, glutathione, and specifically the iron-sulfur (Fe-S) group. 25

*Candida albicans* biofilms induced by glucose show a biofilm thickness that has more cell composition than its extracellular polysaccharides. It has been explained by Santana et al 17 that glucose can significantly increase cell numbers and metabolic activity in the early stages of biofilm development. This is not in accordance with research conducted by Jung et al 26 that the addition of glucose or sucrose shows an increase in extracellular polysaccharide production on biofilms.

*Candida albicans* biofilm induced by lactose has a thickness that is not much different from glucose induction because lactose is composed of glucose and galactose monosaccharides. Biofilm growth by galactose induction is slower than glucose induction so that the thickness of the biofilm formed is slightly thinner compared to glucose induction alone. 27

*Candida albicans* biofilm induced by soy protein can be formed due to the presence of specific carbohydrate-binding proteins, namely, lectins, which can bind to glycoproteins, glycolipids, and polysaccharides so that they can mediate biological processes by binding to glucose sources. This specific bond helps EPS to envelop microorganisms. 28

*Candida albicans* biofilms induced by iron provide the thickest biofilm thickness among other inducers. *Candida albicans* absorbs metal ions easily thereby increasing the formation of biofilms in the early stages of microcolonies and during the process of biofilm maturation thereby increasing extracellular polysaccharides on biofilms. Small amounts of iron in the environment can increase polysaccharide production extracellular. This can increase the attachment of cells to the surface. 8 The limitations in this study are the existence of various factors that can affect biofilm bacteria such as pH, O _2_ , signal molecules, and other substances as sources of metabolism, which have not been studied so that further research is needed with these various factors. Various types of inducing factors can influence the various structure of the biofilm because each factor represents a different nutritional requirement for each type of microbe contained in the biofilm. Thus, we must consider the food intake we consume so that it does not trigger the formation of oral bacterial biofilms.

## Conclusion

The chemical elements of *Candida albicans* biofilm induced by glucose, lactose, soy protein, and iron have the same percentage of composition of elements, namely, oxygen, carbon, nitrogen, phosphorus, and the least were sulfur and the thickest biofilm was by the induction of iron, glucose, and lactose, and the thinnest was by soy protein.
